# Causal association of gastroesophageal reflux disease with obstructive sleep apnea and sleep-related phenotypes: a bidirectional two-sample Mendelian randomization study

**DOI:** 10.3389/fneur.2023.1283286

**Published:** 2023-11-29

**Authors:** Shan Qin, Chi Wang, Xiaoqiu Wang, Wenzhong Wu, Chengyong Liu

**Affiliations:** Jiangsu Province Hospital of Chinese Medicine, Affiliated Hospital of Nanjing University of Chinese Medicine, Nanjing, China

**Keywords:** GERD, OSA, sleep-related phenotypes, causality, MR analysis

## Abstract

**Background:**

The interactions and associations between obstructive sleep apnea (OSA), sleep-related phenotypes (SRPs), and gastroesophageal reflux disease (GERD) are complex, thus it is hard to explore the effect and direction of causalities.

**Study objectives:**

A bidirectional Mendelian randomization (MR) study was performed to explore causal associations of GERD with OSA and SRPs (including insomnia, morningness, sleep duration, ease of getting up, daytime napping, daytime dozing, and snoring).

**Methods:**

First, we gathered summary statistics from publicly available databases. Subsequently, we identified single-nucleotide polymorphisms without strong linkage (r^2^ ≤ 0.001) by referencing relevant genome-wide association studies that met genome-wide significance criteria. Our primary analysis relied on inverse variance weighted to estimate the causal relationship. To ensure the validity of our findings, we also conducted several sensitivity analyses. These included MR Pleiotropy RESidual Sum and Outlier to detect and correct for potential pleiotropic effects, MR-Egger to assess directional pleiotropy, and weighted median analysis to further evaluate heterogeneity and pleiotropy. For the initial MR analysis, when causality was indicated by the results, instrumental variables that were significantly linked to the aforementioned confounding factors were removed. We will re-analyze the data after excluding outcome-related single nucleotide polymorphisms to confirm that the results are still consistent with the previous results.

**Results:**

GERD was found to increase the risk of OSA (OR = 1.53, 95% CI = 1.37–1.70, *p* = 5.3 × 10^−15^), insomnia (OR = 1.14, 95% CI = 1.10–1.19, *p* = 1.3 × 10^−10^), snoring (OR = 1.09, 95% CI = 1.04–1.13, *p* = 6.3 × 10^−5^) and less sleep duration (OR = 0.94, 95% CI = 0.91–0.97, *p* = 3.7 × 10^−4^). According to the reverse-direction analysis, there is an elevated risk of GERD associated with OSA (OR = 1.07, 95% CI = 1.02–1.12, *p* = 0.005), insomnia (OR = 1.95, 95% CI = 1.60–2.37, *p* = 1.92 × 10^−11^) and snoring (OR = 1.74, 95% CI = 1.37–2.21, *p* = 4.4 × 10^−6^).

**Conclusion:**

Genetic susceptibility to GERD can elevate the likelihood of experiencing insomnia, snoring, and OSA, in addition to diminishing sleep duration. Conversely, a reverse MR analysis indicates that ameliorating any one of insomnia, snoring, or OSA can mitigate the risk of developing GERD.

## Introduction

In recent years, sleep disorders have increasingly become a burden of public health problems, and these patients often exhibit one or more of the following symptoms, including difficulty falling asleep, easy awakenings, shortened sleep duration, early awakening, snoring, and even sleep apnea (1). Among them, sleep apnea is a very common and dangerous but easily ignored symptom (2). Significantly, an estimated 936 million adults aged 30–69 years are afflicted with varying degrees of obstructive sleep apnea (OSA), making it a prevalent and pressing public health concern (3). At present, the management of sleep-related problems in clinical settings is still challenging. In general, just like using positive pressure ventilation to improve sleep apnea, people also use sleeping pills to temporarily alleviate symptoms such as difficulty falling asleep, frequent awakenings, and early awakenings (4). However, none of these methods address the root cause, and they can lead to patients becoming overly dependent on treatment, without fundamentally resolving the issue (5). The high prevalence of OSA and the associated adverse clinical outcomes of OSA (6), including cardiovascular disease occurrence and death (7), suggest the need for more perspective and deeper research to deepen our understanding of OSA and to explore the pathophysiology and underlying risk factors that produce OSA, helping us to develop new prevention and treatment strategies that will positively impact population health and healthcare expenditures.

Gastroesophageal reflux disease (GERD) is defined as the effortless flow of gastric contents into the esophagus or mouth (8), resulting in discomforting symptoms or complications, including sleep issues (9). Kim et al. (10) found that GERD was associated with OSA as well as deterioration in sleep quality. A recent investigation (11) has demonstrated that GERD was significantly related to OSA, even after controlling for potentially confounding variables such as age, gender, ethnicity, obesity, asthma, other respiratory diseases, as well as sinus and throat diseases. Nevertheless, the validity of this pathophysiological mechanism has been challenged in certain investigations. For instance, Kuribayashi et al. (12) found no association between the severity of OSA or negative intraesophageal pressure resulting from OSA and the development of sleep-related GERD or reflux esophagitis. However, a meta-analysis with a higher level of evidence that included 2,699 patients found a significant relationship between OSA and GERD (13). Out of the 11,685 survey participants who had GERD, 88.9% reported experiencing symptoms during the night, 68.3% had trouble with their sleep, 49.1% found it hard to fall asleep, and 58.3% had difficulty staying asleep. Those who had nighttime GERD symptoms were more likely to encounter sleep problems, as well as difficulties when trying to fall asleep and stay asleep (14). Another cross-sectional case–control study, encompassing 65,333 participants, suggests that the association between sleep issues and gastroesophageal reflux disease may be bidirectional (15). To summarize, the association between GERD and OSA has been a subject of relatively limited controversy, and the same holds true for GERD in relation to sleep-related phenotypes (SRPs) (including insomnia, morningness, sleep duration, ease of getting up, daytime napping, daytime dozing, and snoring). But their causal link remains uncertain due to the inadequacy of high-quality evidence and the complexity of assessing bidirectional causal associations in conventional epidemiological studies.

Mendelian Randomization (MR) is a pivotal method for resolving this perplexity, utilizing single nucleotide polymorphisms (SNPs) as instrumental variables (IVs) to assess the true causal relationship between exposure and outcomes through genetic variations (16). Consequently, the causal relationships between GERD and OSA, as well as GERD and SRPs, can be further elucidated.

## Method

### Data sources for OSA

We utilized publicly available summary statistics from the OpenGWAS website^1^ for OSA traits among 217,955 individuals of European ancestry. The dataset, identified as finn-b-G6_SLEEPAPNO, comprised 16,761 cases of sleep apnea and 201,194 control cases, which were obtained from Finland’s nationwide health registries. The diagnosis of OSA was made in accordance with the International Classification of Diseases (ICD-10: G47.3, ICD-9: 3472A), based on subjective symptoms, clinical examination, and sleep registration utilizing the apnea-hypopnea index (AHI) ≥5/h or respiratory event index (REI) ≥5/h (17).

The preliminary screening and harmonized analysis data are presented in Supplementary Tables S3, S4, respectively. Outliers and SNPs associated with the outcome are indicated by red and blue markers. More details of the genome-wide association data set used in this study are presented in Supplementary Table S1.

### Data sources for SRPs

The summary statistics for insomnia were derived from a large-scale genome-wide association studies (GWAS) comprising a vast sample size of 1,331,010 individuals (18). Genetic associations with insomnia were downloaded from the publicly available GWAS among individuals of European ancestry contributed from CTGlab.^2^ We obtained summary statistics for insomnia (*n* = 386,533), morningness (*n* = 345,552), sleep duration (*n* = 384,317), ease of getting up (*n* = 385,949), daytime napping (*n* = 386,577), daytime dozing (*n* = 386,548), and snoring (*n* = 359,916) from the UK Biobank due to access constraints to 23andMe samples (*n* = 944,477). More details about the trait definition are available in Supplementary Table S1.

### Data sources for GERD

The summary data for GERD were derived from the largest published GWAS study on gastroesophageal reflux in European populations, comprising a sample size of 602,604 individuals, of which 129,080 were cases and 473,524 were controls. And, the GWAS summary-level data on GRED is available through OpenGWAS (see text footnote 1). Controls in this study were defined as individuals without any history or current occurrence of upper digestive system disorders. GERD cases were defined based on a combination of self-reported GERD symptoms such as heartburn, the use of GERD medication, and hospital records based on ICD-10 codes (19).

### Study design

**Figure 1:**
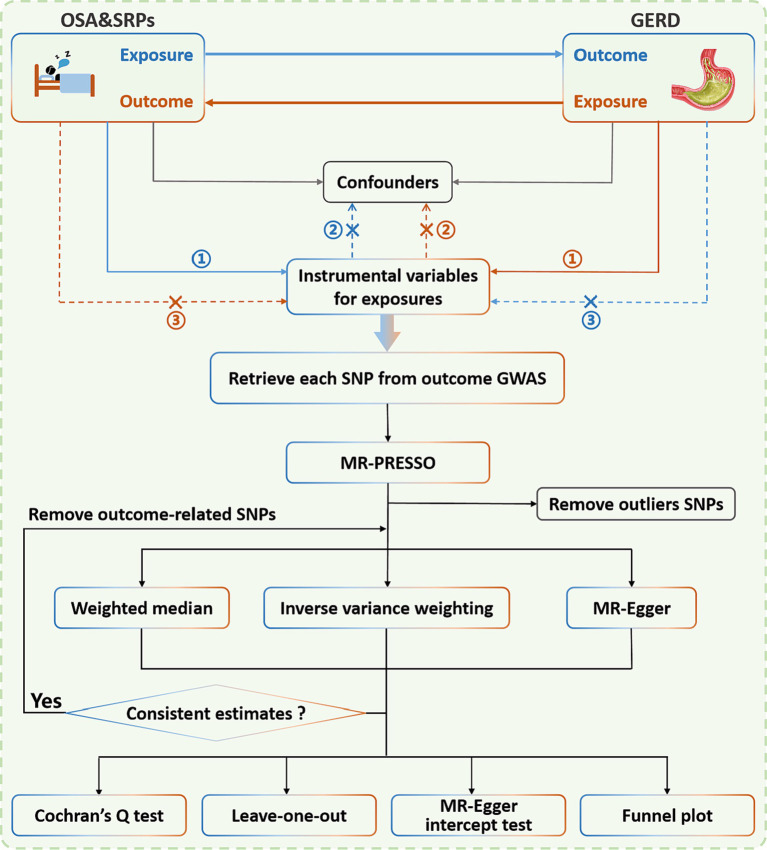
Illustration portraying the bidirectional study employing the Mendelian randomization approach to draw causal inferences linking OSA, SRPs, and GERD. It is worth noting that the Mendelian randomization approach is founded upon three fundamental assumptions: ① the instrumental variables (IVs) have a substantial correlation with the exposure; ② the IVs are unrelated to any confounding variables; and ③ the IVs affect the outcome solely through the exposure, and not by any other means. The blue line illustrates the Mendelian randomization analysis of the connection between OSA/SRPs and GERD. Meanwhile, the brown line represents the Mendelian randomization analysis of the connection between GERD and OSA/SRPs. OSA, obstructive sleep apnea; SRPs, sleep-related phenotypes; GERD, gastroesophageal reflux disease; SNP, single nucleotide polymorphism; GWAS, genome-wide association study; MR-PRESSO, MR-pleiotropy residual sum and outlier.

This is a two-sample bidirectional Mendelian randomized study to assess the potential bidirectional causal associations of GERD with OSA and SRPs. The three hypotheses of the MR Study design include: (1) correlation hypothesis: single-nucleotide polymorphism (SNP) is strongly correlated with exposure; (2) exclusivity hypothesis: SNP is not associated with the outcome; (3) independence hypothesis: the SNP does not appear to be associated with confounding factors (20). SNPs that were strongly associated with exposure (*p* < 5 × 10^−8^) were used as instrumental variables in most cases in this study. To include more SNPs contributing to GERD, we used a more lenient threshold (*p* < 5 × 10^−6^) in studies with OSA, daytime dozing, daytime napping, and insomnia as outcomes; this has also been used previously in many psychiatric MR studies (21, 22). Simultaneously, to mitigate the potential bias due to the linkage disequilibrium (LD) relationship among SNPs, we employed the clump data function within the TwoSampleMR package and restricted the physical distance between SNPs to be >10,000 kb, while ensuring the R^2^ of LD between genes to be <0.001. After removing the outliers identified with MR Pleiotropy RESidual Sum and Outlier (MR-PRESSO) and outcome-related SNPs, the instrumental variable is finally obtained. The schematic diagram depicted in Figure 1 offers a comprehensive overview of the study framework. It’s important to emphasize that SRPs are simply a collective term for several sleep-related symptoms. During Mendelian randomization analysis, each of these symptoms or characteristics is treated individually as either exposures or outcomes.

### Statistical analysis

In this MR analysis, we use three different methods to investigate the relationship between genetic variants and a specific outcome, including random-effect inverse-variance weighted (IVW), MR Egger, and weighted median. To account for variant heterogeneity and pleiotropy, we removed the outcome-related SNPs and outliers that were identified using MR-PRESSO.

The IVW method was selected as the primary method for outcome analysis, while MR-Egger and the weighted median method were employed to enhance the IVW estimates. MR-Egger enables all genetic variants to possess pleiotropic effects but necessitates that the pleiotropic effects be unrelated to the variant-exposure association (23). The weighted median method was utilized in the MR analysis, which allows the inclusion of genetic instruments with potentially invalid causal effects on the exposure of interest. This approach assumes that a minimum of half of the instruments used in the analysis are valid, thereby reducing the potential bias introduced by invalid instruments on the overall effect estimate. To assess the presence of heterogeneity in the MR analysis, we employed Cochran’s Q test, which examines the variability across the genetic variants used in the analysis. A significant Q statistic indicates the presence of heterogeneity, which could potentially bias the MR results. Additionally, we used the funnel plot to visually inspect for asymmetry, which may indicate the presence of directional pleiotropy. To evaluate the presence of horizontal pleiotropy, we used the MR-Egger intercept test, which examines whether genetic variants have pleiotropic effects that violate the MR assumptions. A significant intercept value indicates the presence of horizontal pleiotropy. Moreover, we performed a leave-one-out analysis to assess the robustness of the MR results by iteratively excluding one genetic variant from the analysis and recalculating the effect estimate. The analyses were conducted using the R package TwoSampleMR (version 0.5.6), with a two-sided *p*-value of 0.05 set as the significance threshold for the global-level test and a Bonferroni correction applied for region-level analyses, where a *p*-value of 0.05/8 was the threshold for significance. And, nominally significant results were defined as *p* < 0.05.

When the results of the preliminary analysis indicate the existence of a causal relationship, we will check in PhenoScanner^3^ to see whether the SNPs were associated with potential confounders and removed SNPs associated with any of these potential confounders (Supplementary Table S2) at genome-wide significance (24, 25). Our focus is on factors associated with GERD, such as obesity and smoking. For the initial MR analysis, when causality was indicated by the results, IVs that were significantly linked to the aforementioned confounding factors were removed. We will re-analyze the data after excluding outcome-related SNPs to confirm that the results are still consistent with the previous results.

We calculate the F-statistics of instrumental variables one by one through the formulas *R*^2^ = 2*(1−EAF)*EAF*β^2^ and *F* = *R*^2^*(*N*−2)/(1−*R*^2^) (*N* is the sample size, and *R*^2^ is the exposure variance explained by the selected SNPs), taking *F* = 10 as the threshold.

## Results

In total, after removing outliers, the number of the detail index SNPs selected to genetically predict GERD is from 57 to 74 (Supplementary Table S3). And, the details of index SNPs selected to genetically predict sleep apnea and SRPs are listed in Supplementary Table S4 (sleep apnea:16, daytime dozing:7, ease of getting up:20, insomnia:37, morningness:26, daytime napping:6, sleep duration:11, snoring:9). The *F*-statistics of these genetic instruments surpassed the commonly adopted threshold value of 10, suggesting their robustness and strength (Supplementary Tables S3, S4). For positive outcomes, SNP effect sizes for exposure were visualized in scatter plots for the outcome (Figures 3A,D,G; Figures 4A,D,G), while leave-one-out sensitivity analysis suggested no individual SNP significantly influenced the association between exposure and outcome (Figures 3B,E,H,K; Figures 4B,E,H), and funnel plots revealed no significant heterogeneity among selected independent variables (Figures 3C,F,I,L; Figures 4C,F,I).

### The causal association between OSA and GERD

#### OSA as the outcome

**Figure 2:**
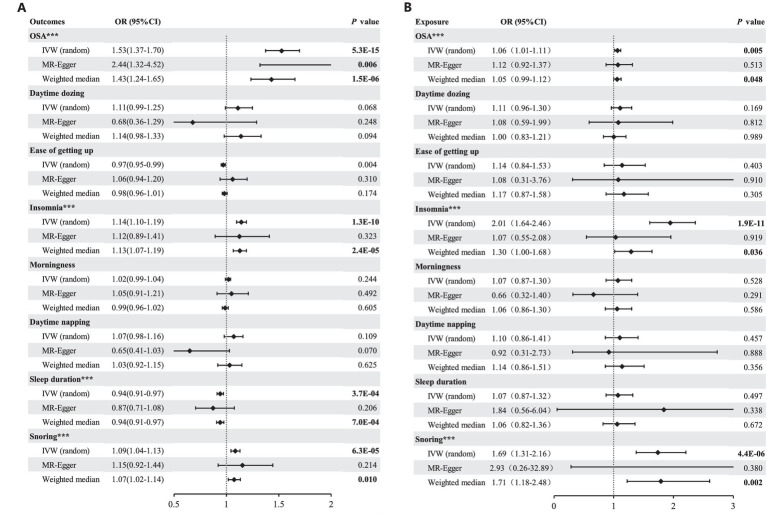
**(A)** Forest plot of Mendelian randomization analysis with OSA and SRPs as outcomes after removing the outliers identified with MRPRESSO; **(B)** Forest plot of Mendelian randomization analysis with OSA and SRPs as exposures after removing the outliers identified with MRPRESSO. ***Significant estimate is defined as IVW-derived *p* < 0.00625. IVW, inverse variance weighted; OR, odds ratio; CI, confidence interval; MR, Mendelian randomization.

**Figure 3:**
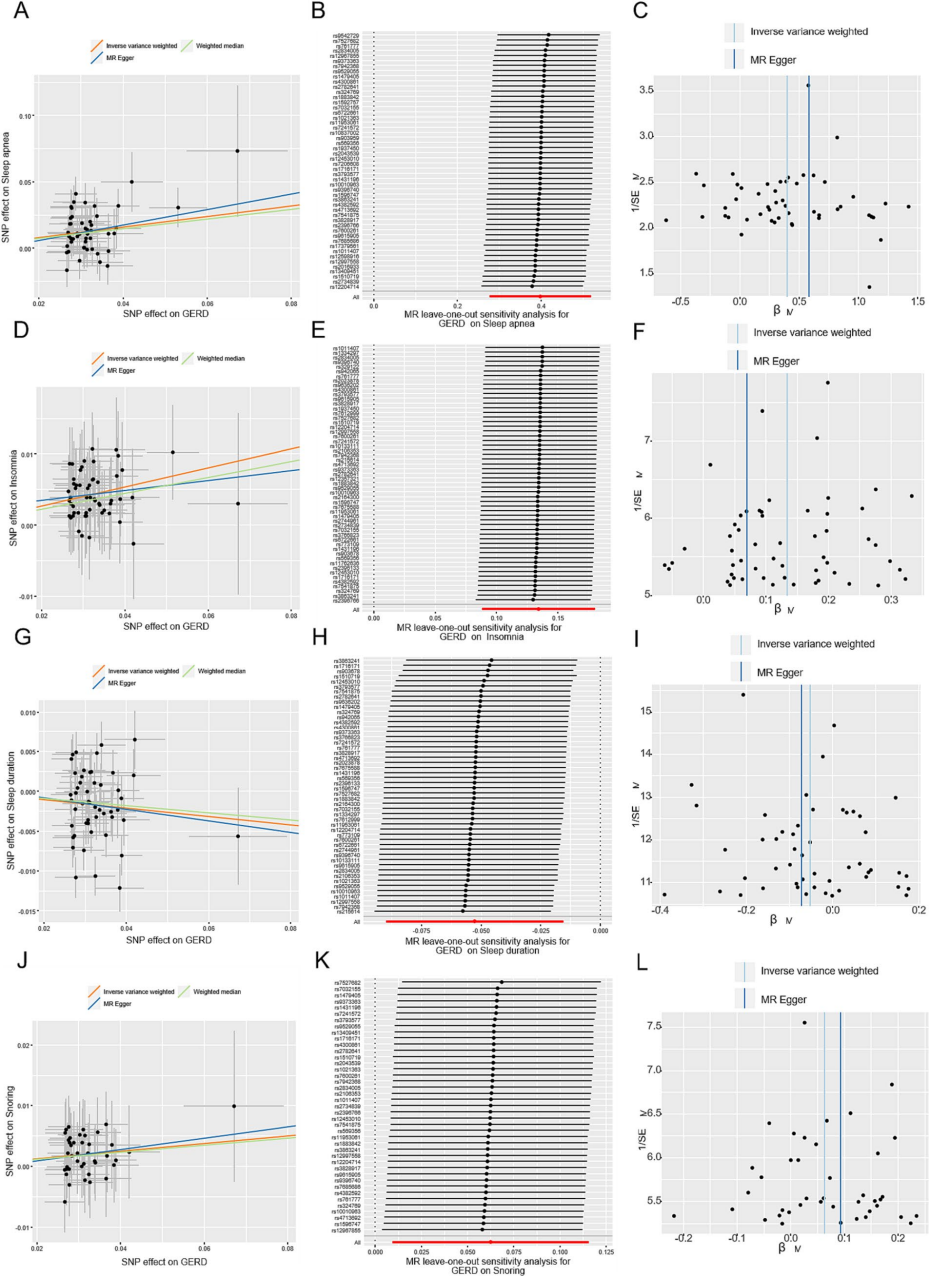
MR-Egger estimates of significant results from GERD on OSA, insomnia, sleep duration, and snoring after removing the outliers identified with MRPRESSO and outcome-related SNPs. **(A,D,G,J)** Scatter plots from genetically predicted GERD on OSA, insomnia, sleep duration, and snoring; **(B,E,H,K)** Leave-one-out plot from genetically predicted GERD on OSA, insomnia, sleep duration, and snoring; **(C,F,I,L)** Funnel plot from genetically predicted GERD on OSA, insomnia, sleep duration, and snoring.

Following correction for a single outlier SNP, a positive causal relationship between genetically predicted GERD and OSA was detected utilizing the IVW method (OR = 1.53, 95% CI = 1.37–1.70, *p* = 5.3 × 10^−15^) (Figure 2A). Consistent estimates of the effect were also obtained using the weighted-median method (OR = 1.43, 95% CI = 1.24–1.65, *p* = 1.5 × 10^−6^). In addition, the sensitivity analysis of MR-Egger gained a similar result (OR = 2.44, 95% CI = 1.32–4.52, *p* = 0.006). And, the MR-Egger regression analysis indicated the absence of directional pleiotropy (*p* = 0.13). After removing the SNPs associated with secondary traits, the adjusted MR analysis did not show any evidence of pleiotropic effects (*p* = 0.66), and the results from the IVW method (OR = 1.49, 95% CI = 1.32–1.68, *p* = 1.6 × 10^−10^) and weight-median method (OR = 1.44, 95% CI = 1.21–1.72, *p* = 4.6 × 10^−5^) corroborated the unadjusted MR analysis (Supplementary Figure S1). SNP effect sizes for GERD were visualized in scatter plots for OSA (Figure 3A).

#### OSA as the exposure

Genetically predicted OSA showed evidence of a causal association with GERD (IVW: OR = 1.07, 95% CI = 1.02–1.12, *p* = 0.005) (Figure 2B). The weighted-median method yielded a comparable estimate (OR = 1.06, 95% CI = 1.00–1.12, *p* = 0.048). The exclusion of secondary trait SNPs did not affect the estimated causal effect of OSA on the risk of GERD (OR = 1.06, 95% CI = 1.01–1.11, *p* = 0.014), as shown in Supplementary Figure S2. Furthermore, the MR-Egger regression analysis indicated no evidence of pleiotropic effects (*p* = 0.57) (Supplementary Table S5:4).

### The causal association between insomnia and GERD

#### Insomnia as the outcome

As shown in Figure 2A, genetically predicted GERD was associated with a higher risk of insomnia (OR = 1.14, 95% CI = 1.10–1.19, *p* = 1.3 × 10^−10^). The weighted-median method provided a consistent estimate (OR = 1.13, 95% CI = 1.07–1.19, *p* = 2.4 × 10^−5^). The potential pleiotropic effect was not found (*p* = 0.87) (Supplementary Table S5:1). Upon exclusion of secondary trait SNPs, the estimate for the causal effect of GERD on the risk of insomnia remained unchanged (OR = 1.14, 95% CI = 1.09–1.20, *p* = 1.7 × 10^–8^), and was consistent with the estimate obtained using the weighted-median method (OR = 1.12, 95% CI = 1.05–1.19, *p* = 2.5 × 10^−4^). Furthermore, the MR-Egger regression analysis yielded a potentially pleiotropy-free estimate (*p* = 0.67) (Supplementary Table S5:3).

#### Insomnia as the exposure

Using the IVW method on results from 37 SNPs, we observed a positive association between genetically predicted insomnia and GERD (OR = 1.95, 95% CI = 1.60–2.37, *p* = 1.9 × 10^−11^). The effect estimate from the weighted-median method was consistent (OR = 1.29, 95% CI = 1.02–1.64, *p* = 0.036). Observing heterogeneity with a Cochran’s Q test derived *p* value <0.05, we deemed it acceptable as we used the random-effects IVW as the main result (26). Meanwhile, pleiotropy was found in the MR-Egger regression analysis (*p* = 0.0497) (Supplementary Table S5:2). Instead, the adjusted MR analysis showed no evidence of potential pleiotropic effects (*p* = 0.06). The corrected estimate after excluding the secondary traits SNPs was consistent (OR = 2.01, 95% CI = 1.64–2.46, *p* = 1.9 × 10^−11^).

### The causal association between snoring and GERD

#### Snoring as the outcome

A positive causal effect of genetic predisposition to GERD on snoring risk was observed (Figure 2A), with an odds ratio of 1.09 per genetically predicted 1-unit increase in log-transformed OR of GERD (95% CI = 1.04–1.13, *p* = 6.3 × 10^−5^). The effects were validated as consistent in the following sensitivity analyses (Weighted median: OR = 1.07, 95% CI = 1.02–1.14*, p* = 0.01). The MR-PRESSO method did not detect any genetic variant outliers, and even after removing the pleiotropic genetic variant identified in the PhenoScanner database, the significant result remained unchanged. The OR of snoring was 1.21 (OR = 1.69, 95% CI = 1.31–2.16, *p* = 4.2 × 10^−5^) per 1-unit increase in log-transformed OR of GERD. The sensitivity analyses of the weighted median yielded robust and consistent results. Furthermore, according to the leave-one-out analysis, none of the single SNPs drove a significant result (Figure 3H).

#### Snoring as the exposure

**Figure 4:**
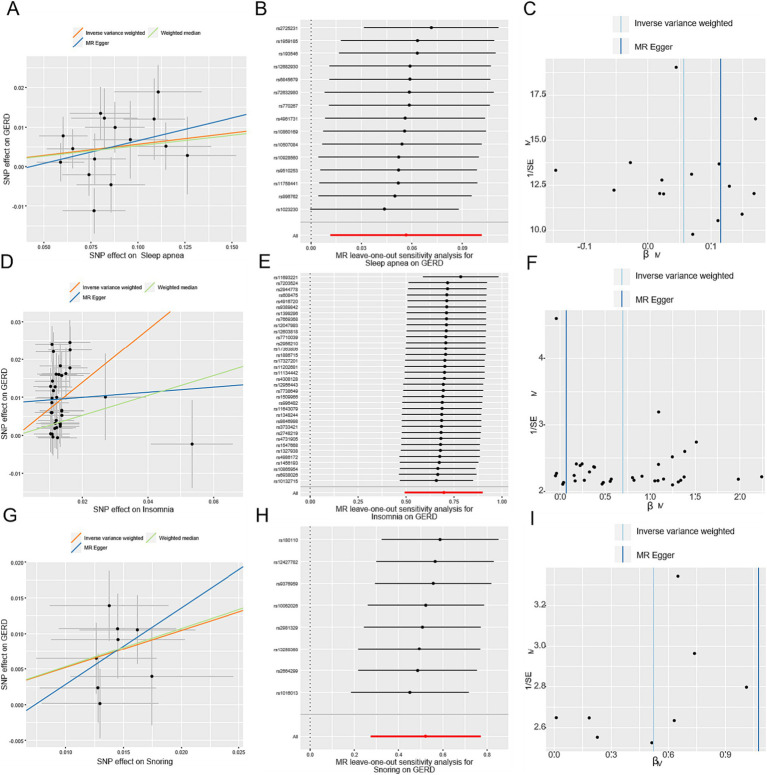
MR-Egger estimates of significant results from OSA, insomnia, and snoring on GERD after removing the outliers identified with MRPRESSO and outcome-related SNPs. **(A,D,G)** Scatter plots from genetically predicted OSA, insomnia, and snoring on GERD; **(B,E,H)** Leave-one-out plot from genetically predicted OSA, insomnia, and snoring on GERD; **(C,F,I)** Funnel plot from genetically predicted OSA, insomnia, and snoring on GERD.

Eleven SNPs were used as instruments in the two-sample MR analyses, which showed a causal effect of genetically predicted snoring on the risk of GERD (Supplementary Table S4:8). The random-effect IVW models showed that genetically predicted snoring was associated with an increased risk of GERD (OR = 1.74, 95% CI = 1.37–2.21; *p* = 4.4 × 10^−6^). Similar results were observed using the weighted median method (OR = 1.79, 95% CI = 1.23–2.60; *p* = 0.002) in sensitivity analyses. Associations of each variant with snoring and the risk of GERD are shown in Figure 2B. There was no evidence of heterogeneity in the IVW analysis (Q = 6.42, *p* = 0.60). MR-Egger regression analysis did not reveal any evidence of directional pleiotropic effects among the genetic variants used as instruments (intercept = −0.009; se = 0.018; *p* = 0.64). Besides, the OR of GERD was OR = 1.69 (95% CI = 1.31–2.16, *p* = 4.2 × 10^−5^) per 1-unit increase in log-transformed OR of snoring after excluding the pleiotropic genetic variant (rs2307111, rs592333 and rs7005777) identified in the PhenoScanner database. As shown in Figure 4H, based on the leave-one-out sensitivity analysis, no single SNP significantly influenced the association between OSA and snoring.

### The causal association between sleep duration and GERD

#### Sleep duration as the outcome

After screening the summary statistics of the GERD GWAS and removing 9 outliers, 57 SNPs were identified and selected as IVs for the subsequent MR analysis (Supplementary Table S3:7). Mendelian randomization analysis using the TwoSampleMR package showed that GERD had a causal relationship with sleep duration, as indicated by the IVW analysis (OR = 0.94, 95% CI = 0.91–0.97; *p* = 3.7 × 10^−4^). Also, the weighted median analysis also demonstrated a causal relationship between GERD and sleep duration (OR = 0.94, 95% CI = 0.91–0.97; *p* = 7.0 × 10^−4^). Horizontal pleiotropy analysis showed that there was no horizontal pleiotropy between sleep duration and the occurrence of GERD (*p* = 0.47). The significant result was not changed by the removal of the pleiotropic genetic variant. The leave-one-out sensitivity analysis demonstrated that the effect of GERD on sleep duration was not substantially influenced by any individual SNP (Figure 3K), indicating the stability of our analysis. Moreover, the funnel plots and forest plots indicated no significant heterogeneity among the selected IVs (Figure 3L).

#### Sleep duration as exposure

Reverse MR analyses using the IVW method did not find a causal association between genetic liability to sleep duration and GERD (OR = 1.07, 95% CI = 0.87–1.32; *p* = 0.497). The absence of an association persisted when using the weighted median and MR-Egger methods as well (Supplementary Table S5:2).

### The causal association between daytime dozing, ease of getting up, morningness, daytime napping, and GERD

We did not find causal relationships between daytime dozing, ease of getting up, morningness, daytime napping, and GERD in either direction (Supplementary Table S5:1, 2).

## Discussion

In our bidirectional MR analysis, we have discovered a bidirectional causation linking OSA with three sleep-related traits and GERD. Our positive Mendelian randomization study indicates a positive correlation between genetically predicted GERD and the probability of experiencing OSA, insomnia, and snoring. Moreover, genetically predicted GERD exhibits a negative association with sleep duration. This finding is consistent with the majority of observational studies that have investigated the relationship between GERD and sleep-related disorders. Notably, a national study involving over 22 million patients revealed that 12.21% of GERD-diagnosed individuals were also diagnosed with OSA, while only 4.79% of non-GERD patients exhibited OSA, thereby establishing a higher OSA prevalence in patients with GERD than in their non-GERD counterparts (27). Additionally, a separate investigation demonstrated that patients with erosive esophagitis manifested more abnormal OSA-related sleep indices than those with GERD symptoms but with normal upper gastrointestinal microscopy (10). Interestingly, two polysomnography monitoring studies further substantiated the GERD-OSA relationship, with one finding that high reflux questionnaire scores increased the risk of OSA (28), and the other discovering that sleep disruption occurred after 76% of acid reflux events (29). A survey shows that 61.7% of patients in France reported experiencing regular (at least once a week) sleep disturbances related to GERD (30). And a cohort study indicates that individuals with GERD have a higher risk of experiencing sleep disturbances compared to those without GERD (31).

Bidirectional MR analysis revealed a positive causal impact of OSA, insomnia, and snoring on GERD, which is in agreement with previous research. The prevalence of Barrett’s esophagus was found to be three times higher in patients with OSA than in those without OSA (32). A large cross-sectional study involving 6,939 individuals revealed that those who experienced fewer than eight sleep awakenings per month were almost 50% less likely to suffer from heartburn and acid reflux compared to those who frequently woke up at night (33). Furthermore, both GERD patients and healthy controls exhibited significantly prolonged esophageal acid exposure after sleep deprivation, according to research (34). An analysis conducted on 19,864 adult individuals in Japan found that poor sleep quality is a significant risk factor for GERD (35). And a prospective study involving 2,316 adult individuals indicates that insomnia is associated with an increased risk of GERD (36). Additionally, a study conducted by Emilsson et al. found that objectively measured snoring was more common in night-GERD subjects than in controls (37). The finding that our study corroborates using MR analysis to show that this association is likely causal.

Numerous concomitant mechanisms corroborate the reciprocal association between OSA and GERD. Inflammation and irritation caused by microaspiration of gastric contents may lead to mucosal swelling and spasms of the upper airway, extending to surrounding soft tissues such as the vocal cords, vocal folds, and laryngeal cartilage, which may increase the thickness of the airway wall (38), or cause bronchial constriction by stimulating the vagus nerve (39), ultimately resulting in snoring sounds or even sleep apnea when severe. While previous research has found no significant differences in pharyngeal dimensions between snorers and non-snorers (40), the study subjects did not include snorers with concomitant GERD. Therefore, it is worth conducting further targeted research to investigate whether pharyngeal dimensions during sleep are abnormal in such a population. Additionally, acid reflux may stimulate the vagus nerve, inhibiting the medullary respiratory center and preventing proper contraction of the respiratory muscles (41), which may also lead to apnea. From a sleep position perspective, the supine position not only facilitates airway obstruction due to tongue and throat tissue collapse (42) but also increases the likelihood of gastroesophageal reflux (43), which can exacerbate respiratory issues by causing breathing pauses and snoring. A study on the relationship between renalase and OSA found that more severe OSA patients often have more shallow sleep (44), which may also help explain why GERD can simultaneously lead to OSA, insomnia, and subjectively shortened sleep duration.

Patients with sleep apnea exhibit a higher probability of reflux due to inadequate respiration, which results in more pronounced intra-thoracic pressure fluctuations. Moreover, apnea and hypoventilation cause a decline in intrathoracic pressure and an increase in intra-abdominal pressure, which exacerbates the degree of esophageal hiatal hernia (45) and, in turn, aggravates gastroesophageal reflux. In addition, oxygen deficiency and carbon dioxide retention caused by apnea and hypoventilation lower intracellular adenosine triphosphate (ATP) levels, which, in turn, decrease the influx of calcium ions into muscle cells and increase tissue acidity (46). These changes, in turn, decelerate the contraction and peristalsis of the gastrointestinal tract and the relaxation of the lower sphincter of the esophagus, increasing the likelihood of gastric contents refluxing into the esophagus (47). Additionally, patients suffering from sleep apnea may experience an increase in both superior vena cava pressure and thoracic negative pressure due to breathing difficulties. This could lead to an increased risk of esophageal varices and a decreased amplitude of peristaltic waves, resulting in delayed food digestion and a further likelihood of gastroesophageal reflux (48). Through our MR study, we have discovered that snoring may impact GERD. This finding underscores the role of genetic factors in this relationship. The discovery owes itself to our utilization of eight specific genes (AC002539.2, LINC00598, SIM1, ADGRV1, UNC5D, LACTB2, BCL11B, C9orf3) as instrumental variables, all associated with snoring. This observation emphasizes the need to not only explore the causality between snoring and gastroesophageal reflux but also investigate how genetic factors mediate or alter this connection. Further research may assist in unveiling potential biological mechanisms and provide a better understanding of the influence of snoring on gastroesophageal reflux, thereby enhancing interventions and treatments.

From a neural perspective, insomnia caused by GERD may be attributed to the interplay of various neural pathways. The excitement of the vagus nerve caused by gastroesophageal reflux may lead to dysfunction in the hypothalamus and amygdala (49–51), two brain regions involved in regulating sleep stability, emotion, and cognition. This can result in unstable sleep and increased susceptibility to external factors that disturb sleep. Additionally, GERD may lead to psychological and emotional discomfort, such as anxiety, fear, and stress (52, 53). These responses may be transmitted through neural circuits in the amygdala, further affecting sleep quality and duration. GERD may also impact the cortico-hypothalamic-brainstem-spinal cord reflex pathway, a crucial neural pathway regulating physiological functions like respiration, circulation, and sleep (54). Irritation from GERD can disturb these physiological responses and affect sleep stability and quality through a similar pathway. In summary, GERD disrupts sleep maintenance by influencing the interaction of multiple neural circuits, including the vagus nerve, hypothalamus, amygdala, and cortico-hypothalamic-brainstem-spinal cord reflex pathways. Abnormal interactions between these neural loops contribute to the effects of GERD on sleep quality, leading to significant decreases in total sleep duration and insomnia.

When suffering from insomnia, tossing and turning in bed are often accompanied by heightened levels of stress and anxiety, which can activate emotion-regulating areas located in the cerebral cortex, such as the amygdala (55). These areas can release neurotransmitters like glutamate, which in turn can activate the hypothalamic–pituitary–adrenal axis and release stress hormones like corticosteroids that affect vagal and sympathetic nerve activity (56). Moreover, the hypothalamus can directly act on the vagal nucleus through the hypothalamic-vagal nucleus pathway, thus increasing its excitability (57). These pathways can interact in multiple ways, thereby decreasing vagal activity. Difficulties in sleep maintenance can also lead to disturbances in the body’s biological clock, which can affect the rhythmic activity of the vagus nerve. The hypothalamic thermoregulatory center controls the body’s biological clock, and insomnia and sleep deprivation can disrupt the thermoregulatory rhythm, thereby affecting vagal nerve activity (58). Additionally, chronic insomnia can lead to altered neuroplasticity, which can increase vagal activity (59). The vagus nerve is a crucial neural regulatory system, and chronic insomnia can alter its activity pattern, which may explain the promoting effect of sleep deprivation on esophageal hypersensitivity and gastroesophageal acid exposure (60). In addition to affecting body temperature changes, circadian rhythm also influences gastrointestinal physiology and melatonin secretion. Moreover, researchers have found that serum melatonin levels in patients with GERD are lower than those in the general population (61). Furthermore, it has been discovered that the selective melatonin receptor agonist Ramelteon can bind to Melatonin Receptor 1(MT1) and Melatonin Receptor 2(MT2) receptors in the suprachiasmatic nucleus and reduce heartburn symptoms, improve sleep efficiency, and shorten sleep latency (62). On the other hand, the utilization of benzodiazepine medications is closely associated with the manifestation of nocturnal heartburn symptoms, plausibly as a result of their impact on the basal pressure of the lower esophageal sphincter, the rate of transient lower esophageal sphincter relaxation, and the motility of the stomach (63). Therefore, for patients with comorbid insomnia and GERD, benzodiazepine drugs should not be recommended over melatonin. Inflammation factors also play an important role in the relationship between insomnia and GERD. Studies have shown that sleep deprivation can lead to the excessive daytime secretion of IL-6 (64), and the levels of inflammatory factors such as IL-1β, IL-2, IL-6, IL-10, and TNF-α are positively correlated with the severity of insomnia (65). Other studies have also shown that the expression of inflammatory factors such as TNF-α, IL-6, IL-1β, and IL-10 is closely related to GERD, and reducing its inflammatory response can have an effective therapeutic effect (66). Another potential mechanism is that, compared to normal sleep, sleep deprivation can alter lifestyle. Sleep deprivation can cause an increase in ghrelin (hunger hormone) levels and a decrease in leptin (satiety hormone) levels, thereby increasing appetite (34). Insomnia provides more awake time, and patients can use this time to further eat, which may lead to more GERD.

### Strengths and limitations

To our knowledge, this represents the inaugural bidirectional MR inquiry simultaneously scrutinizing the causal nexus amid GERD and OSA, alongside GERD and seven sleep-related phenotypes. Given the latent pleiotropy of numerous SNPs, rigorous endeavors have been undertaken to obviate the potential influences of weak instrumental variables. The robustness of the findings in this study has been substantiated through multifarious sensitivity analyses. The salient advantage of MR lies in its capacity to mitigate the pernicious biases engendered by residual confounding (67), owing to the stochastic nature of genetic variations. It is noteworthy that some studies have postulated a plausible causal relationship between GERD and OSA, which might be confounded by common risk factors such as obesity (12). However, our MR analysis has meticulously excluded SNPs that could be influenced by factors like obesity and smoking, while still buttressing the antecedently posited causal linkage. Since genetic variations remain impervious to the vicissitudes of disease states, this obviates the potentiality of reverse causality in the analysis. The lucidity of the ascertained causal relationships is poised to foster enhanced prognostication and preventive strategies for related maladies, furthering the realm of precision medicine. Nevertheless, this study has certain limitations. Firstly, the number of instrumental variables used was limited, and the screening criteria were relaxed in some cases during the reverse MR analysis (*p* < 5 × 10^−6^). Secondly, our conclusions were based on European ancestry and may not apply to other populations. Additionally, according to Dickman’s viewpoints (68), there are significant differences in the features of GERD during wakefulness and sleep, so GWAS data on GERD classified based on sleep and wake subtypes will be more helpful in exploring the causal relationship more precisely. Furthermore, in the GWAS data of this study, the definition of insomnia includes two categories: difficulty in falling asleep and easy to wake up. There is a bidirectional causal relationship between GERD and insomnia, but whether it is more closely related to difficulty in falling asleep or easy to wake up is still uncertain. More detailed subtype classification will be more helpful to explore the causal relationship and potential mechanism. Finally, sleep features are currently classified subjectively through questionnaires, and the extraction and classification of objective sleep electroencephalography features are time-consuming, so relevant GWAS data are not yet available. Therefore, the causal relationship between subtypes of sleep stage-based classification and GERD and other diseases cannot yet be refined. We also believe that with the rapid development and widespread use of portable wearable sleep monitoring devices, we will obtain more objective and accurate sleep data from larger samples in the future, allowing for more valuable Mendelian randomization analyses.

## Conclusion

This bidirectional Mendelian randomization study found GERD can elevate the likelihood of experiencing insomnia, snoring, and OSA, in addition to diminishing sleep duration. Conversely, ameliorating any one of insomnia, snoring, or OSA can mitigate the risk of developing GERD.

## Data availability statement

The original contributions presented in the study are included in the article/Supplementary material, further inquiries can be directed to the corresponding authors.

## Ethics statement

All the genes used in the research data are from the public database in the large-scale genome-wide association study (GWAS), so the study does not require ethical approval.

## Author contributions

SQ: Conceptualization, Data curation, Investigation, Writing – original draft. CW: Formal Analysis, Methodology, Resources, Visualization, Writing – original draft. XW: Writing – review & editing. WW: Writing – review & editing. CL: Writing – review & editing.

## Footnotes

^1^ https://gwas.mrcieu.ac.uk/

^2^ https://ctg.cncr.nl/software/summary_statistics

^3^ www.phenoscanner.medschl.cam.ac.uk
