# Corrigendum: Causal association of gastroesophageal reflux disease with obstructive sleep apnea and sleep-related phenotypes: a bidirectional two-sample Mendelian randomization study

**DOI:** 10.3389/fneur.2024.1441003

**Published:** 2024-06-11

**Authors:** Shan Qin, Chi Wang, Xiaoqiu Wang, Wenzhong Wu, Chengyong Liu

**Affiliations:** Jiangsu Province Hospital of Chinese Medicine, Affiliated Hospital of Nanjing University of Chinese Medicine, Nanjing, China

**Keywords:** GERD, OSA, sleep-related phenotypes, causality, MR analysis

In the published article, there was an error in the Funding statement. The correct project name for Grant Number SJCX23_0869 should be “Postgraduate Research & Practice Innovation Program of Jiangsu Province,” rather than the currently listed “Graduate Research and Innovation Projects of Jiangsu Province.” The correct Funding statement appears below.

