# Supplementary material for: Causal association of gastroesophageal reflux disease with obstructive sleep apnea and sleep-related phenotypes: a bidirectional two-sample Mendelian randomization study
Source: Front Neurol. 2023 Nov 29;14:1283286. doi: 10.3389/fneur.2023.1283286 (PMC10716286; doi:10.3389/fneur.2023.1283286)

**Supplementary figure 1**Forest plot of Mendelian randomization analysis with OSA and SRPs as outcomes after removing the outliers identified with MRPRESSO and outcome-related SNPs. IVW, inverse variance weighted; OR,odds ratio; Cl, confidence interval; MR, Mendelian randomization.


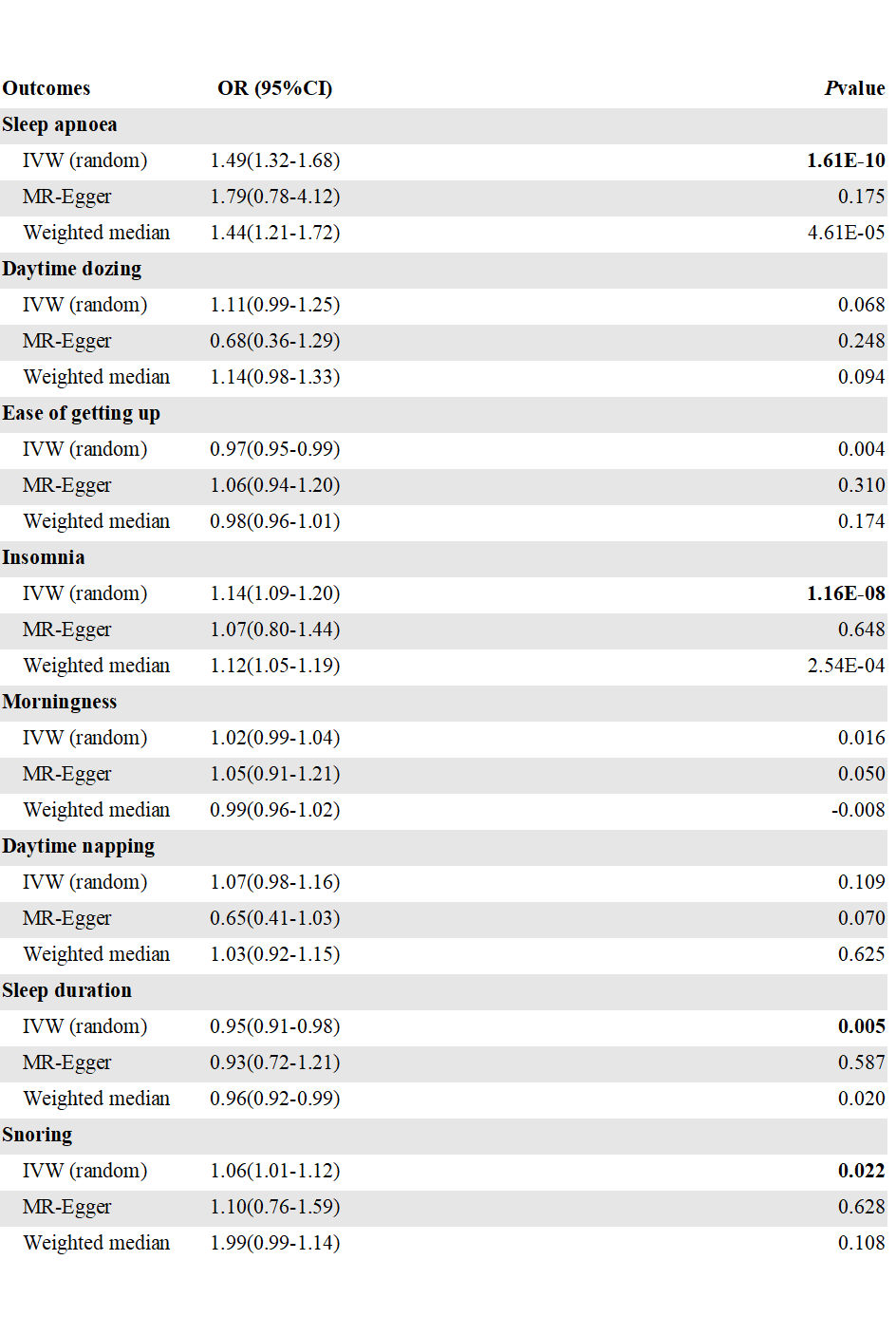

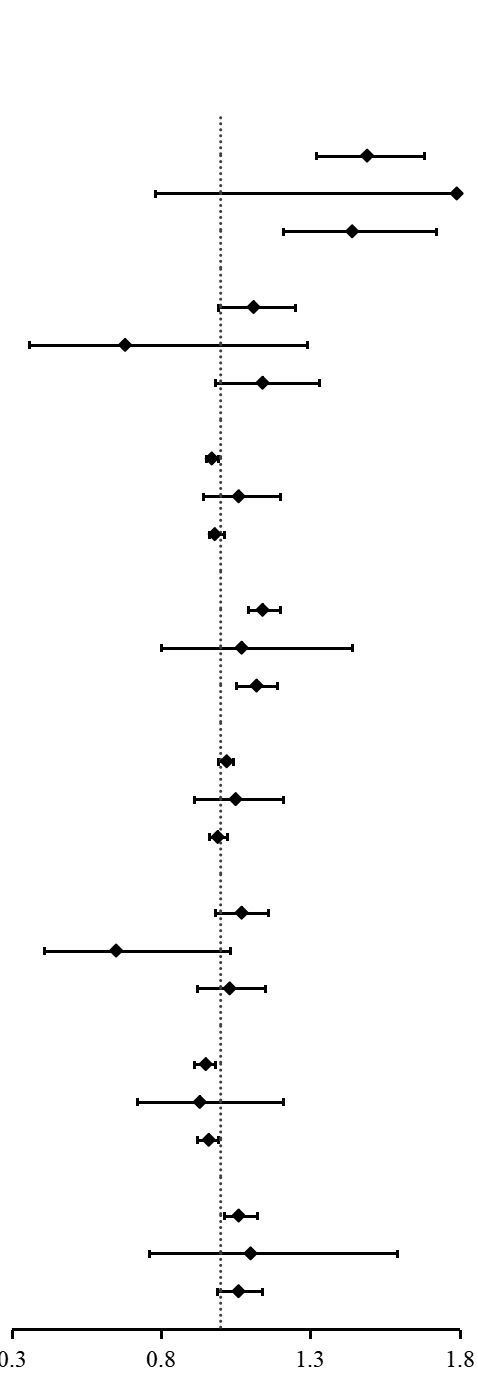


**Supplementary figure 2**Forest plot of Mendelian randomization analysis with OSA and SRPs as exposures after removing the outliers identified with MRPRESSO and outcome-related SNPs. IVW, inverse variance weighted; OR,odds ratio; Cl, confidence interval; MR, Mendelian randomization.


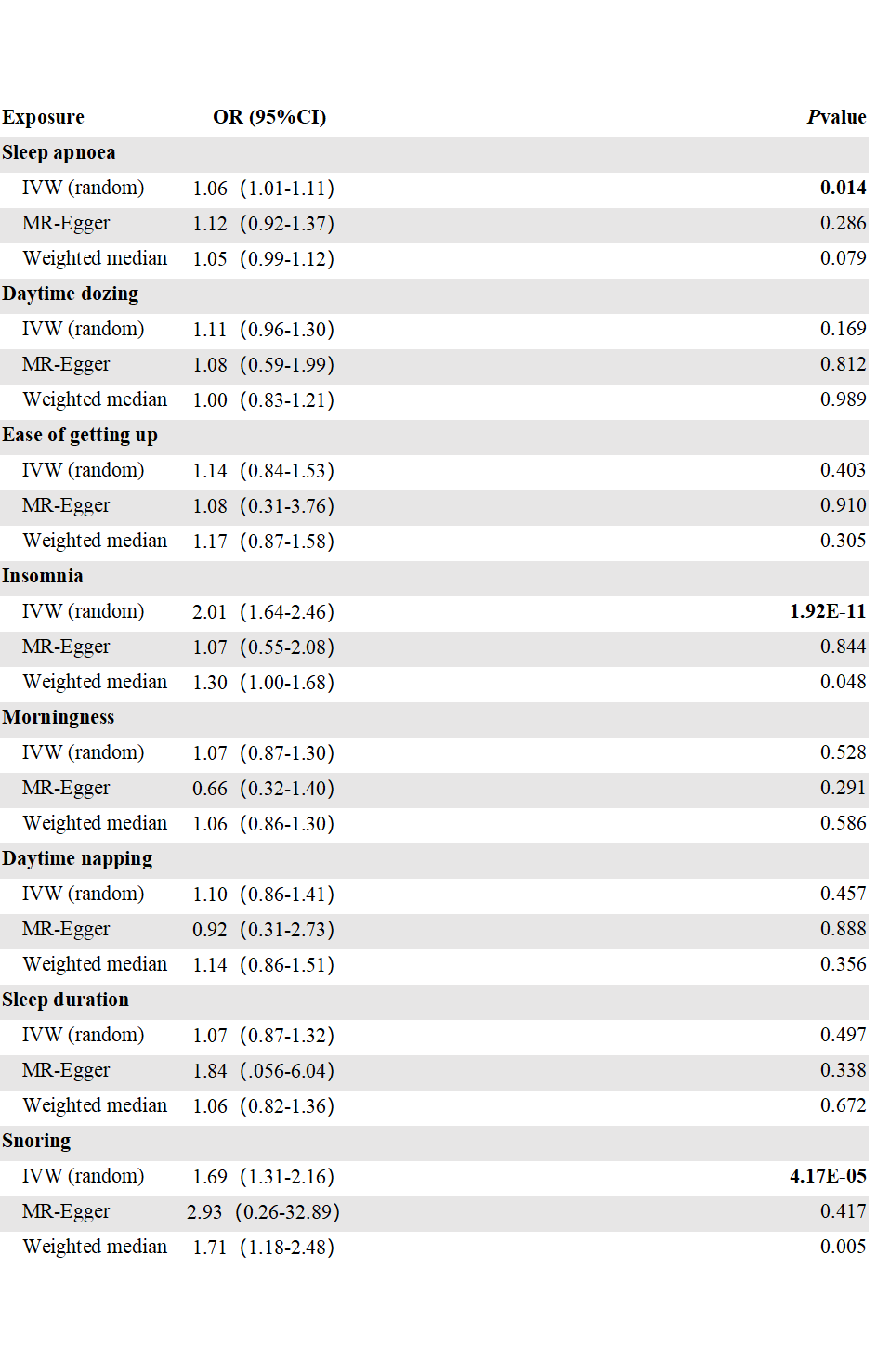

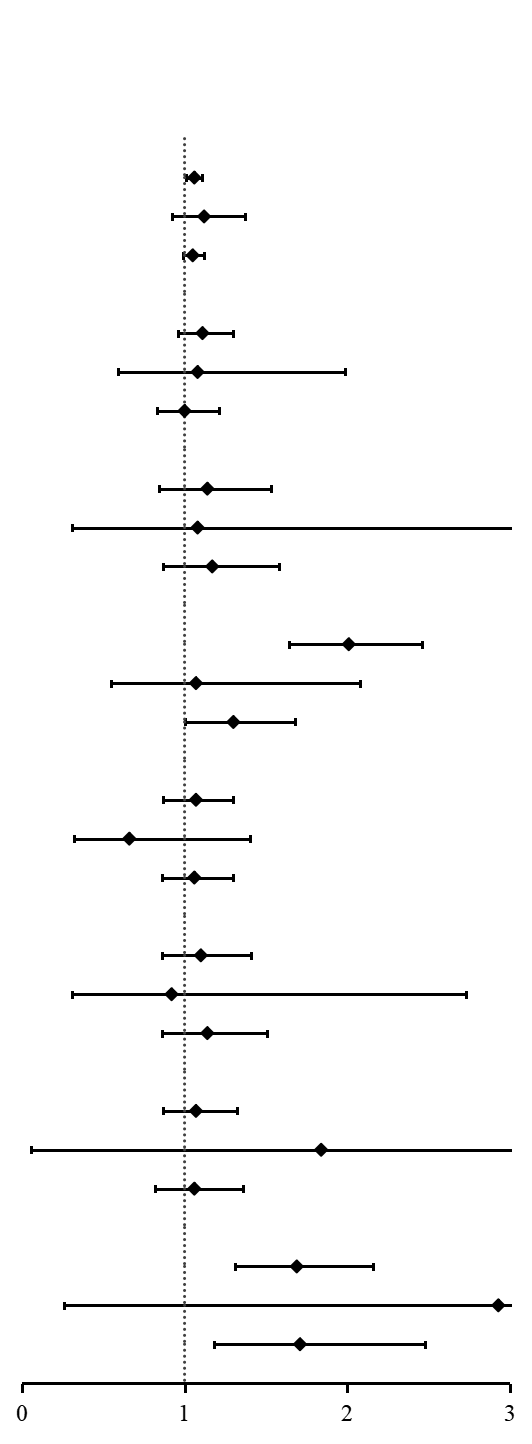

Supplement: Supplementary file 6 [file Data_Sheet_6.docx]
